# Perceived cognitive functioning and its influence on emotional vulnerability in breast cancer

**DOI:** 10.1177/2055102919871661

**Published:** 2019-08-23

**Authors:** Bethany Chapman, Stefanie Helmrath, Nazanin Derakshan

**Affiliations:** University of London, UK

**Keywords:** anxiety, cognitive function, depression, emotional vulnerability, quality of life

## Abstract

We investigated the relationship between perceived cognitive function and emotional vulnerability of breast cancer survivors while examining the moderating effect of various risk factors. Results confirmed that perceived cognitive function predicted emotional vulnerability with grade of breast cancer moderating this relationship. Age at diagnosis exhibited a trend towards significance for emotional vulnerability, and time since diagnosis as well as grade significantly predicted quality of life. Our findings imply that (younger) women with a higher breast cancer grade are at a greater risk for emotional and cognitive vulnerability and can benefit from interventions designed to reduce emotional vulnerability through training cognitive efficiency.

## Introduction

Breast cancer is the most prevalently diagnosed malignancy among women (Breast Cancer Care, 2019), with a new diagnosis being given every 10 minutes in the United Kingdom (Breast Cancer Care, 2018). While continuous improvements in the anti-cancer treatments offered for breast cancer have positively increased the number of women living into long-term survivorship (95.8% of women survive at least 1 year and 85.3% survive 5 years) (Office for National Statistics, 2019), research has shown that receiving a breast cancer diagnosis and undergoing a programme of active anti-cancer treatment induce a series of largely negative physical, psychological, emotional and cognitive short-term and long-term side-effects or impairments (Bower, 2008; Janelsins et al., 2011; Philip et al., 2013).

Interestingly, strong evidence has linked systemic chemotherapy treatment to cognitive dysfunction in around 16–75 per cent of breast cancer survivors, a phenomenon commonly known as the ‘chemo-brain’ (Bower, 2008; Janelsins et al., 2014). In particular, survivors frequently reported experiencing difficulties in a range of cognitive domains including short- and long-term memory, executive function, attention, information processing and language production and/or comprehension (Cheung et al., 2012; Janelsins et al., 2011, 2014; Myers, 2013). Endocrine therapy medications such as Tamoxifen and Aromatase Inhibitors (AI) have also been found to independently cause extensive levels of cognitive dysfunction (Bakoyiannis et al., 2016; Bender et al., 2015). Concerningly, researchers have identified that these impairments in cognitive functions can persist for many years after the completion of anti-cancer treatment (Ahles et al., 2012; Koppelmans et al., 2012) significantly impairing quality of life (Klemp et al., 2018; Von Ah et al., 2009).

A majority of women following the diagnosis of breast cancer are at greater risk of developing depression, anxiety and post-traumatic stress disorder (PTSD) (Baqutayan, 2012; Jacob et al., 2016; Kenyon et al., 2014; Maass et al., 2015; Mehnert and Koch, 2007; Tsaras et al., 2018), which adversely impact quality of life and psychosocial well-being (Akel et al., 2017; Dragomir et al., 2013; Saeedi-Saedi et al., 2015) as well as the ability to function in everyday situations (i.e. working ability) (Adler and Page, 2008). It is critical that we develop a stronger understanding of the predictive power of the key risk factors (demographic factors, and perceived cognitive function) influencing emotional vulnerability, to aid the implementation of more targeted therapies for sustained emotional resilience.

A considerable body of research has explored the effects of key breast cancer demographic factors (risk factors) in relation to women’s emotional health. For example, Van Londen et al. (2014) showed that breast cancer survivors actively receiving endocrine therapy medications (i.e. Tamoxifen) expressed higher levels of emotional distress, more potent worries about the possibility of cancer recurrence and greater upset regarding their appearance. Such negative emotional symptomologies are thought to be partly explained by the role of endocrine therapy medication on the natural production of oestrogen, noradrenaline and serotonin (5-HT) (Ito et al., 2006). Physical side effects including hot flushes, menopause, fatigue and thromboembolic events (Kilickap et al., 2013; Syrowatka et al., 2017) are also likely to be linked to these heightened emotions. In addition, researchers have revealed that endocrine therapy medications significantly increase the severity of cognitive impairment experienced and lower women’s physical health–related quality of life (Ganz et al., 2016).

The grade of breast cancer diagnosed has only recently been investigated as a possible factor influencing quality of life of survivors. Despite long-standing evidence that a higher grade is associated with a greater chance of premature death (Elston and Ellis, 1991) and more intense anti-cancer treatment(s), interestingly, Yang et al. (2017) identified higher grades (i.e. grade 2 (moderate) or 3 (high)) of cancer to be independently associated with a higher risk of experiencing emotional symptomologies including anxiety and depression. One possible explanation is that women with high grades have more persistent ruminative thinking regarding cancer recurrence, metastasis and premature death. Considering that grade appears to have such a high emotional impact on breast cancer survivors, it is essential to address this factor in this study to ensure positive developments in the field and current interventions.

Furthermore, it has been reported that women diagnosed at a younger age exhibit a greater level of emotional distress (Gabriel and Domchek, 2010; Levkovich et al., 2018; Yang et al., 2017) during the post-treatment period compared to older women. This has been associated with an acute long-term fear of developing secondary cancer and recurrence or metastasis of the original breast cancer (Wan et al., 2016; Ziner et al., 2012), as women diagnosed at a younger age often have more aggressive forms of cancer including invasive histopathology, greater node involvement, high grade, larger tumour size, increased human epidermal growth receptor 2 (HER2) and negative oestrogen receptors (Copson et al., 2013; Gajdos et al., 2000; Kheirelseid et al., 2011; Yazdani-Charati et al., 2019). Foreseeably, studies also delineated that worries regarding the symptoms of early menopause including hot flushes, night sweats and vaginal dryness (Rosenberg and Partridge, 2013) as well as fertility incapacity, parenting responsibilities, work duties and finance are highly powerful catalysts elevating emotional symptoms in young women diagnosed with breast cancer (Gabriel and Domchek, 2010; Ganz et al., 2003; Ruddy et al., 2013; Sharma and Purkayastha, 2017; Wan et al., 2018).

It is clear from recent evidence that women experience the most severe levels of emotional vulnerability straight after the diagnosis and immediately after the completion of active anti-cancer treatment (Arving et al., 2007; Burgess et al., 2005; Henselmans et al., 2010). Although emotional stability progressively improves over time (Andersen et al., 2017; Burgess et al., 2005), the risk of developing emotional symptomologies including depression, anxiety and PTSD can remain atypically high for many years (Andersen et al., 2017; Bleiker et al., 2000).

While a substantial proportion of evidence has alluded to the existence of a noteworthy bidirectional relationship between the cognitive function and emotional well-being of breast cancer survivors (Von Ah et al., 2013; Von Ah and Tallman, 2015), only a scarce amount of research has examined this relationship systematically (Von Ah and Tallman, 2015). Implementing the Functional Assessment of Cancer Therapy–Cognitive Scale (FACT-Cog) to assess 88 post-treatment breast cancer survivors, Von Ah and Tallman (2015) revealed that perceived cognitive impairments, as well as perceived cognitive abilities, were significantly associated with the level of depression and anxiety experienced supporting a strong interplay between perceived cognitive function and the emotional disorders experienced by breast cancer survivors.

The concept that cognitive flexibility plays a determining role in maintaining a healthy emotional well-being has received increasing support from recent research that has successfully targeted cognitive dysfunction(s) using cognitive control training (CCT) to boost emotional well-being in depression (Koster et al., 2017; Motter et al., 2016), as well as in subclinical anxiety and worry (Course-Choi et al., 2017; Sari et al., 2016). Accordingly, cognitive control has also been treated as a protective mechanism against emotional vulnerability impeding the progression of psychological distress to clinical psychopathology. Such research demonstrates that CCT can be used to improve cognitive decline and, most importantly, depicts that the functions of cognition may protect against emotional vulnerability.

A recent training study conducted by Swainston and Derakshan (2018) revealed that breast cancer survivors who received 12 sessions of online dual n-back training experienced sustained improvements in emotional symptomologies including rumination, anxiety and depression up to 15 months after the completion of training. Previous research has also found cognitive memory and speed-processing training among this population to result in improvements of symptom distress and quality of life (Von Ah et al., 2012). While novel, these findings are highly pertinent given the high amount of psychological (or emotional) distress experienced pre-diagnosis, during and beyond anti-cancer treatment.

The current study investigated the relationship between breast cancer survivor’s perceived cognitive function and their perceived emotional vulnerability and examined the predictive power and moderating role of four key demographic factors including (1) age at diagnosis, (2) time since diagnosis, (3) endocrine therapy status and (4) grade of breast cancer in this relationship, as well as in relation to the survivor’s quality of life. We elected to analyse these four particular demographic factors, as research has consistently pinpointed them to be highly salient influencers on breast cancer survivor’s emotional health and/or quality of life. Based on current research, we predicted that perceived cognitive function would significantly relate to perceived emotional vulnerability and quality of life. We also predicted that the four demographic (risk) factors would be associated with the severity of emotional vulnerability reported and moderate the relationship between survivor’s perceived cognitive function and their perceived emotional vulnerability.

## Method

### Design

The design was cross-sectional. Participants were instructed to complete a battery of six online questionnaires on cognitive and emotional health. The first variable we measured was the participant’s perceived cognitive function; this was measured at two levels through the execution of the FACT-Cog and Perception of Cognition Questionnaire (PCQ). The second variable measured was the participant’s perceived emotional well-being; we measured this on four levels through the implementation of one mainstream questionnaire (Hospital Anxiety and Depression Scale (HADS)) and three cancer-specific questionnaires (Revised Impact of Events Scale (IES-R), Quality of Life Questionnaire (QoL) and Cancer Worry Scale (CWS)).

### Participants

The participants recruited to partake in the present research were women (*N* = 132, mean age = 48.86, *SD* = 8.84; mean age at diagnosis = 45.58, *SD* = 8.41; mean number of months since diagnosis = 39.95, *SD* = 34.80) (one participant failed to specify their diagnosis as primary or secondary) who had previously received a clinical diagnosis of either primary (*n* = 119, mean age = 49.05, *SD* = 8.71; mean age at diagnosis = 46.08, *SD* = 8.21; mean number of months since diagnosis = 36.34, *SD* = 26.68) or secondary (*n* = 12, mean age = 47.08, *SD* = 10.60; mean age at secondary diagnosis = 40.67, *SD* = 9.57; mean number of months since diagnosis = 77.50, *SD* = 71.14) breast cancer.

Participants had to be in a post-active treatment phase not receiving anti-cancer treatment(s) including chemotherapy and/or radiotherapy. The participant could, however, be administering a regular hormone blocker therapy medication (i.e. Tamoxifen) or receiving target treatments (i.e. Herceptin injection). Participants with a current or former diagnosis of either a neurological or psychiatric condition (i.e. anxiety or depression) were eligible to take part.

Participants were recruited using voluntary sampling through online advertisements placed on social media platforms including the ‘Building Resilience in Breast Cancer Centre’ (BRiC Centre; http://briccentre.bbk.ac.uk/). This research received ethical approval from the Research Ethics Committee of the Department of Psychological Sciences at Birkbeck College, University of London, and in accordance with the guidelines, all of our participants provided informed consent before completion of the study.

### Materials

#### The General Demographics Questionnaire (GDQ)

The 29-item GDQ was developed to provide demographics such as the participants’ breast cancer diagnosis (i.e. primary or secondary breast cancer), clinical characteristics of their tumour (i.e. severity/grade of tumour, lymph node involvement, and hormone receptor status), and the programme of active anti-cancer treatment they received (i.e. systemic chemotherapy and/or surgical). In addition, the GDQ also collects information relating to the general health habits (i.e. smoking, alcohol intake), neurological and psychiatric medical history.

#### FACT-Cog

The FACT-Cog, Version 3 (Wagner et al., 2009) consists of 37 positively or negatively phrased items that evaluate participants’ subjective understanding of the treatment-related changes in their cognitive function and the impact that these cognitive changes have had on their perceived quality of life in the last 7 days. The items are sectioned into four subscales (perceived cognitive impairments, comments from others, perceived cognitive abilities and impact on quality of life). Higher scores illustrate better cognitive function.

#### PCQ

The PCQ (Galantino et al., 2006) is a 7-item self-report chemotherapy specific questionnaire that assesses the perceived changes in the cognitive function(s) of breast cancer patients following the completion of chemotherapy. The first six items known as the ‘perception of cognition’ measure the extent of cognitive change experienced, while the last item probes the general quality of life. Each item is measured on a 7-point Likert-type scale with higher scores indicating better cognitive function.

#### The IES-R for cancer care

The IES-R is a standardised questionnaire by Horowitz et al. (1979) that can be modified for application in cancer care (Weiss, 2007; Weiss and Marmar, 1997). The modified IES-R consists of 22 tailored items measuring cancer-related thoughts. It reflects the *Diagnostic and Statistical Manual of Mental Disorders* (4th ed.; *DSM-IV*) criteria for PTSD and assesses three key symptoms including avoidance, hyperarousal and intrusion. Individual’s responses are based on their own personal experience(s) with the symptoms over the last 7 days, with a higher combined score reflecting a greater PTSD symptom severity.

#### HADS

The HADS (Zigmond and Snaith, 1983) is a broadly used scale that measures levels of anxiety and depression among many clinical populations. It consists of 14 items: 7 items relating to anxiety and 7 items relating to depression. Individual’s responses are based on the feelings (emotions) experienced over the past 7 days, with higher scores indicating more chronic levels of depression and/or anxiety.

#### The QoL Short Version

The shortened QoL questionnaire (Ferrell et al., 1995) includes a 25-item, ordinal scale (ranging from 0 to 10) measuring the quality of life of breast cancer patients. Subdivided into two scales known as ‘physical health well-being’ (i.e. fatigue) and ‘psychological well-being’ (i.e. life satisfaction), the QoL contains a series of questions addressing the individual’s experience(s) with distress throughout the illness and treatment period with higher scores representing better quality of life.

#### CWS

The CWS (derived from Custers et al.’s (2014) original questionnaire) is an 8-item self-report questionnaire that measures the severity of fear for cancer recurrence and the impact that this fear has on the day-to-day functioning of individuals following treatment. Items are rated on a 4-point Likert-type scale, with ‘1’ indicating ‘not at all or rarely’ and ‘4’ representing ‘almost all the time’. Higher scores indicate higher fear of cancer recurrence.

### Procedure

Upon voluntary request, participants were emailed an information document outlining the primary purpose of the research as well as a secure web-address link (URL code) that re-directed them to the battery of self-report questionnaires. Before the study could commence, participants were presented with an online consent form. After the completion of this form, eligible participants were directed to fill in the 29-item GDQ regarding their personal breast cancer history followed by the two cognitive and four emotional well-being questionnaires.

### Statistical analysis

A series of statistical analyses were performed using Statistical Package for the Social Sciences (SPSS, version 24). Pearson’s correlation analyses were conducted between the two cognitive and four emotional well-being questionnaires to examine whether perceived cognitive function related to the emotional symptoms (vulnerability) (i.e. anxiety, depression, PTSD and cancer recurrence worry) and quality of life encountered by breast cancer survivors.

Two hierarchical regression analyses were performed: the first to examine the relationship between the breast cancer–specific demographic factors (i.e. grade of breast cancer), perceived cognitive function and perceived emotional vulnerability (IES-R + HADS + CWS),^1^ and the second to investigate the influential factors on survivors’ perceived quality of life. In step 1 of our regression analyses, we included only the four selected demographic factors: (1) age at diagnosis, (2) time since diagnosis, (3) endocrine therapy status and (4) grade of breast cancer diagnosed. Measures of perceived cognitive function were then added in step 2.

Using analysis of standardised residual, we found no outliers in the data (emotional vulnerability: standard residual minimum = –2.35, standard residual maximum = 2.74; quality of life: standard residual minimum = –2.14, standard residual maximum = 2.87). Checks for violations of the assumptions of collinearity, independent error, normality, homoscedasticity and linearity were also conducted for each of the regression models using residuals (see Supplementary Material 1). In addition, post hoc analyses for achieved statistical power were conducted.

Finally, using moderation analyses, we investigated the moderating roles of the four aforementioned demographics on perceived cognitive function (as measured by the FACT-Cog questionnaire) in predicting perceived emotional vulnerability (IES-R + HADS +CWS), as well as quality of life. Mean-centred values for perceived cognitive function and quality of life were used. Checks for violations of the assumption of heteroscedasticity were also carried out, and all standard errors in the model were based on the Heteroscedasticity-Consistent Standard Error (HC3) estimator.

We decided to use the FACT-Cog total score in place of the four subscales for perceived cognitive function to increase power in our analyses.

## Results

**Figure 1.**
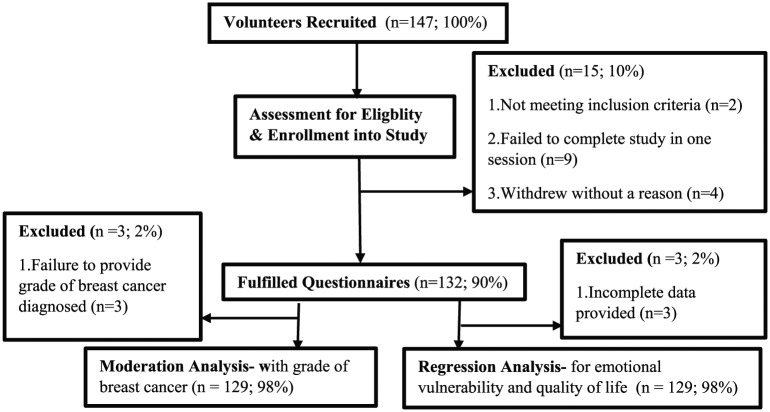
Flowchart of the breast cancer survivor’s eligibility for the present study.

Of the 147 breast cancer survivors who originally volunteered to participate in our study, only two participants failed to meet the initial inclusion criteria (both were receiving active anti-cancer treatment). A further nine eligible participants, however, failed to complete the seven questionnaires during a single session, and four participants withdrew from the study without providing a reason (see Figure 1). Three additional participants were removed from the current regression analyses and three from the grade of breast cancer moderation analysis due to the incompleteness of the information they provided.

### Correlation analysis

Perceived cognitive functioning as measured by the FACT-Cog correlated negatively with the three emotional symptomology questionnaires (IES-R, HADS and CWS). These results indicate that higher levels of impaired cognitive functioning were associated with higher levels of emotional vulnerability inclusive of experiencing worse cancer recurrence worries, anxiety, depression, and PTSD symptoms (see Table 1). Similarly, correlations were identified for the PCQ with the IES-R and HADS.

**Table 1.** Correlations between questionnaires measuring cognitive function and emotional vulnerability.

	IES-R	HADS	QoL	CWS	FACT-Cog	PCQ
IES-R	1	.65**	−.56**	.56**	−.42**	−.31**
HADS		1	−.71**	.49**	−.59**	−.42**
QoL			1	−.56**	.58**	.34**
CWS				1	−.25**	*ns*
FACT-Cog					1	.51**
PCQ						1

IES-R: Revised Impact of Events Scale; HADS: Hospital Anxiety and Depression Scale; QoL: Quality of Life Questionnaire; CWS: Cancer Worry Scale; FACT-Cog: Functional Assessment of Cancer Therapy–Cognitive Scale; PCQ: Perception of Cognition Questionnaire.

** Correlation is significant at the .01 level (two-tailed).

Predictably, our analyses also showed strong positive correlations between the QoL questionnaire and both perceived cognitive function questionnaires (FACT-Cog and PCQ) suggesting that a higher perceived cognitive function is associated with a better overall quality of life (see Table 1).

### Regression analysis

Our first regression analysis (see Table 2) showed that the demographic factors in step 1 accounted for only a small 5 per cent of the variance in emotional vulnerability. When FACT-Cog was entered at step 2, the second model explained 26 per cent of the variance in emotional vulnerability significantly, *F*(5, 123) = 8.77, *p* < .001. Of the four demographic factors included, only age at diagnosis exhibited a trend towards statistical significance (*p* = .12) in our final model, and FACT-Cog remained a significant predictor (*p* < .001). These results indicate that cognitive function predicts emotional vulnerability above and beyond the four demographic factors, with lower levels of perceived cognitive function meeting higher levels of perceived emotional vulnerability.

**Table 2.** Hierarchical regression analyses for the predictors of emotional vulnerability and quality of life.

	*b*	*SE B*	*B*	*t*	*p*
Emotional vulnerability					
Step 1					
Constant	2.41 [–0.62, 5.44]	1.53		1.57	.12
Age at diagnosis	−0.06 [–0.11, –0.00]	0.03	−.18	−2.03	.04
Grade of breast cancer	0.27 [–0.37, 0.92]	0.33	.08	0.84	.40
Endocrine therapies	−0.38 [–1.39, 0.64]	0.51	−.07	−0.73	.46
Time since diagnosis	−0.00 [–0.02, 0.01]	0.01	−.02	−0.23	.82
Step 2					
Constant	4.96 [2.15, 7.77]	1.42		3.49	.00
Age at diagnosis	−0.04 [–0.09, 0.01]	0.02	−.13	−1.56	.12
Grade of breast cancer	0.15 [–0.42, 0.72]	0.29	.04	0.53	.60
Endocrine therapies	−0.39 [–1.29, 0.50]	0.45	−.07	−0.87	.39
Time since diagnosis	0.00 [–0.01, 0.01]	0.01	.00	0.02	.98
FACT-Cog Questionnaire	−0.04 [–0.05, –0.02]	0.01	−.47	−5.97	.00
Quality of life					
Step 1					
Constant	117.95 [78.40, 157.50]	19.98		5.90	.00
Age at diagnosis	0.52 [–0.18, 1.22]	0.35	.13	1.47	.15
Grade of breast cancer	−10.09 [–18.40, –1.71]	4.24	−.21	−2.38	.02
Endocrine therapies	0.87 [–12.35, 14.09]	6.68	.01	0.13	.90
Time since diagnosis	−0.14 [–0.31, 0.04]	0.09	−.14	−1.57	.12
Step 2					
Constant	77.04 [43.21, 110.86]	17.09		4.51	.00
Age at diagnosis	0.24 [–0.33, 0.81]	0.29	.06	0.83	.41
Grade of breast cancer	−8.15 [–15.00, –1.29]	3.47	−.17	−2.35	.02
Endocrine therapies	1.14 [–9.65, 11.92]	5.45	.02	0.21	.84
Time since diagnosis	−0.16 [–0.30, –0.02]	0.07	−.16	−2.29	.02
FACT-Cog Questionnaire	0.59 [0.44, 0.73]	0.07	.57	7.96	.00

Emotional vulnerability: *R*^2^ = .049 for step 1, ∆*R*^2^ = .213 for step 2 (95% confidence intervals for *B*); quality of life: *R*^2^ = .083 for step 1, ∆*R*^2^ = .312 for step 2 (95% confidence intervals for *B*). FACT-Cog: Functional Assessment of Cancer Therapy–Cognitive Scale.

Checks for violation of assumptions using residuals revealed that assumptions of collinearity (all tolerance > 0.1, variance inflation factor (VIF) < 10), independent errors (Durbin–Watson value = 1.83), normality and homogeneity of variance and linearity were all met for this regression model (see Supplementary Material 1). Moreover, post hoc analysis showed that this regression had a medium effect size (Cohen’s ƒ^2^ = 0.29) and an achieved statistical power of (1 – ß error probability) = 1.00.

Our second regression analysis investigating the quality of life (see Table 2) disclosed that the four demographics entered on step 1 accounted for a modest 8 per cent of the variance in women’s perceived quality of life.

FACT-Cog explained an extra 31 per cent of the variance at a significant level, *F*(5, 123) = 16.08, *p* < .001. Importantly, both the grade of breast cancer diagnosed (*p* = .02) and the time since diagnosis (*p* = .02) as well as FACT-Cog (p < .001) functioned as significant predictors in the final model. These results show that perceived cognitive function predicts overall quality of life above and beyond that of the four demographic factors included. Specifically, lower perceived function meets a poorer quality of life.

Checks for violation of assumptions using residuals showed that assumptions of collinearity (all tolerance > 0.1, VIF < 10), independent errors (Durbin–Watson value = 1.91), normality and homogeneity of variance and linearity were met in this regression model (see Supplementary Material 1). Post hoc analysis revealed that this regression analysis had a large effect size (Cohen’s ƒ^2^ = 0.52) and an achieved statistical power of (1 – ß error probability) = 1.00.

### Moderation analysis

Our moderation analyses revealed that of the four moderators assessed, grade of breast cancer diagnosed significantly moderated the relationship between breast cancer survivor’s perceived cognitive function (as measured by the FACT-Cog) and their perceived emotional vulnerability. The result indicated that approximately 32 per cent of the variation in the severity of emotional vulnerability reported could be explained by the main effects (grade of breast cancer and cognitive vulnerability) and the interaction effects (grade of breast cancer × cognitive vulnerability) (*R*^2^ = .32, *F*(5, 123) = 10.14, *p* < .001). The *R*^2^ change due to the interaction was significant, *p* = .01. In addition, the analyses also revealed that none of the four demographic factors functioned as significant moderators in the relationship between perceived cognitive function and the quality of life of breast cancer survivors. All of the corresponding standard errors in the models were heteroscedasticity consistent, and thus, the assumption of heteroscedasticity was met.

**Figure 2.**
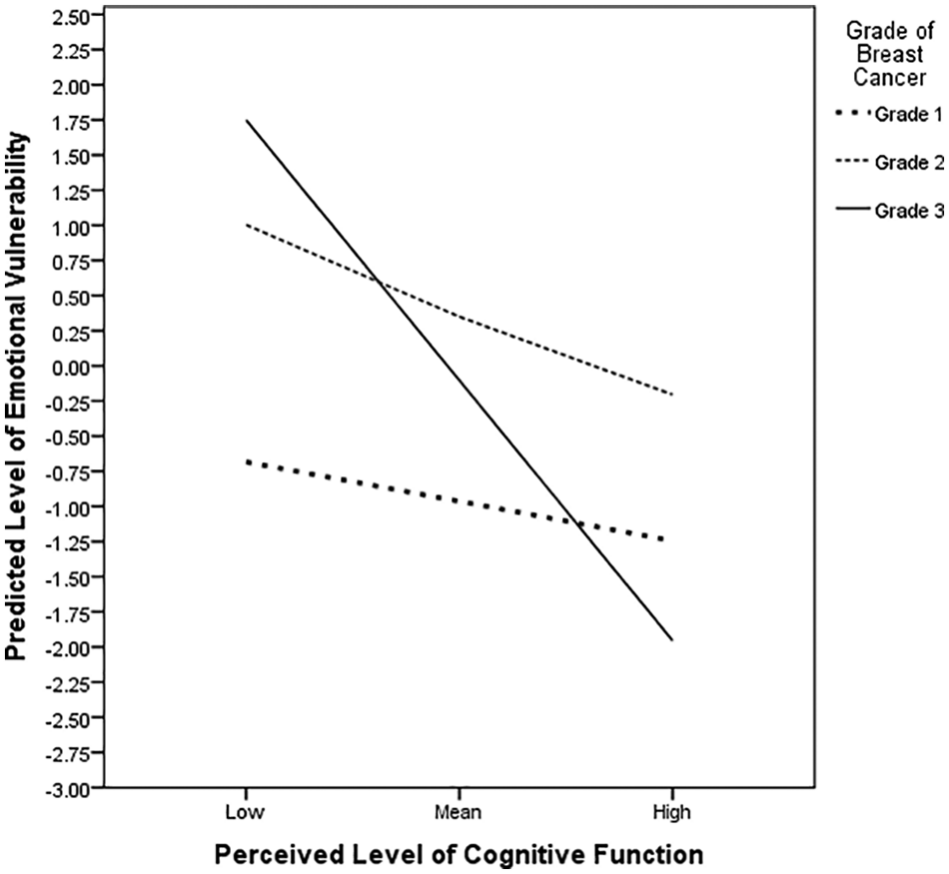
Line chart of simple slope equations for the regression of predicted emotional vulnerability on perceived cognitive ability at three grades of breast cancer.

The line chart of the simple slopes equations in Figure 2 reveals the interaction between perceived cognitive function and emotional vulnerability moderated by grade such that strong negative relationships were found for grades 2 (*p* = .04) and 3 (*p* < .001) (see Table 3) suggesting that the relationship between perceived cognitive function and perceived emotional vulnerability is significantly greater in breast cancer survivors diagnosed with higher (more severe) grades of cancer.

**Table 3.** Conditional effects for grade of breast cancer as a moderator of perceived emotional vulnerability.

Grade of breast cancer	*b*	*SE B*	*t*	*p*
Grade 1	−0.01 [–0.06, 0.04]	0.03	−0.32	.75
Grade 2	−0.02 [–0.04, –0.00]	0.01	−2.10	.04
Grade 3	−0.06 [–0.08, –0.04]	0.01	−6.20	.00

## Discussion

This study investigated the relationship between perceived cognitive function and perceived emotional vulnerability and quality of life in breast cancer with a specific focus on demographic risk factors such as age at diagnosis, time since diagnosis, endocrine therapy status and grade of breast cancer.

As predicted, our results established a link between perceived cognitive functioning and emotional vulnerability as well as quality of life. We found that age at diagnosis showed a trend towards being a significant predictor of emotional vulnerability, while both the grade of breast cancer and the time since diagnosis significantly predicted women’s perceived quality of life. Moreover, our moderation analyses unveiled that women’s grade of breast cancer also significantly moderated the effects of cognitive function in predicting perceived emotional vulnerability.

Our findings on the link between cognitive function and emotional vulnerability extend on Von Ah and Tallman’s (2015) findings and imply that as a women’s confidence in their cognitive function improves and they become more able to live their daily life independently, the level to which they endure anxiety and/or depression symptomology decreases. Advancing the present body of literature, the results also showed perceived cognitive function to be associated with fears of cancer recurrence and PTSD symptomologies, emotions that appear to have been mostly overlooked in previous cognitive breast cancer research. It is important to note, however, that our findings might have been influenced by the inclusion of breast cancer survivors with a current or previous diagnosis of depression (28%) or anxiety (5%). Considering the diverse range of symptoms associated with anxiety and depression (NHS, 2016a, 2016b), further qualitative research is required to develop a better understanding of the specific individual symptoms (i.e. irritability or feelings of hopelessness) affected by cognitive function.

Imperatively, the results from the regression analysis further revealed that breast cancer survivors’ perceived cognitive function was the strongest predictor of their perceived level of emotional vulnerability, with grade of breast cancer significantly moderating this relationship. We found that a higher (more severe) grade resulted in a stronger relationship in which lower perceived cognitive function was coupled with a higher level of emotional vulnerability (see Figure 2). The present study is to our knowledge the first to establish the detrimental effect that grade of breast cancer has on the relationship between survivors’ cognitive and emotional health. Earlier research conducted by Yang et al. (2017) revealed that women with higher histological grades of invasive breast cancer had a greater short-term risk of experiencing anxiety and depression. Such findings are likely caused by higher levels of negative ruminative thinking about cancer recurrence, metastasis and premature death. Our findings highlight the need for professionals to consider women with higher breast cancer grade as more vulnerable and ensure that they are provided with adequate information as well as suitable emotional and cognitive (i.e. CCT) support to reduce the future risk of developing an affective disorder.

In line with previous research, age at the time of breast cancer diagnosis was found to show a strong trend for predicting emotional vulnerability in women, with a younger age inferring higher levels of emotional vulnerability. Notably, our results appear to largely support recent evidence that women diagnosed at a younger age experience more intense emotional distresses (Gabriel and Domchek, 2010; Levkovich et al., 2018; Yang et al., 2017) (such as anxiety and/or depression) elevating their overall level of vulnerability. One possible explanation for this finding is that younger women experience a more persistent and intense fear of cancer recurrence, metastasis and possible premature death (Wan et al., 2016; Ziner et al., 2012), as younger age at diagnosis is often associated with more aggressive forms of cancer including high-grade tumours (Copson et al., 2013; Gajdos et al., 2000; Kheirelseid et al., 2011; Yazdani-Charati et al., 2019). In addition, evidence has revealed that women diagnosed at a younger age experience more intense distress provoking factors including fear regarding the symptoms of early menopause (such as hot flushes, night sweats) (Rosenberg and Partridge, 2013), finance, fertility incapacity and parenting responsibilities (Gabriel and Domchek, 2010; Ganz et al., 2003; Ruddy et al., 2013; Wan et al., 2018), all of which are likely to prolong and enhance levels of emotional distress. Given the disabling effects that anxiety, depression, cancer recurrence worry and PTSD have on survivor’s psychosocial well-being and their ability to function in everyday life (i.e. the ability to attend work) (Adler and Page, 2008; Saeedi-Saedi et al., 2015), this finding could positively direct the field of psycho-oncology to ensure that post-treatment support programmes are tailored to meet the survivor’s individual needs. For example, women diagnosed at a younger age should receive more frequent sessions of post-treatment support to aid a high level of emotional stability, which would, in turn, enable the maintenance of a more ‘typical’ daily routine.

Contradictory to past research, which has reported that the demographic factors such as endocrine therapy (Van Londen et al., 2014) and time since diagnosis (Arving et al., 2007; Burgess et al., 2005; Henselmans et al., 2010) each has a strong effect on the severity of emotional vulnerability endured by breast cancer survivors, this present study found no significant associations between these demographic (risk) factors and the level of emotional vulnerability reported. It could be stipulated that no significant association was found with the effects of time on emotional vulnerability, as the vulnerability is at its highest in the immediate months following diagnosis and active anti-cancer treatment (Andersen et al., 2017; Burgess et al., 2005). Given that the mean time since diagnosis was 39.95 months for our study, this effect may have gone unnoticed. Similarly, approximately one-third of the recruited participants were not prescribed endocrine therapy medications such as Tamoxifen as part of their programme of treatment, medications which have been greatly associated with increased risk for developing emotional distress and cancer recurrence worry (Van Londen et al., 2014); this may have lowered our predictive power for this demographic factor. Due to the noteworthy disparity in our research, it would be highly valuable to further replicate this component of the study with a much larger participant sample.

Corresponding with previous research (Klemp et al., 2018; Von Ah et al., 2009), we found that cognitive function was predictive of perceived quality of life. Specifically, the present study identified that women who outlined experiencing less deterioration in their cognitive function concurrently reported having a higher overall quality of life. Grade of breast cancer diagnosed was predictive of quality of life, with a higher grade (i.e. grades 2 or 3) resulting in a lower perceived quality of life. A likely explanation for this result is that women diagnosed with more severe grades of breast cancer endure a greater and more perpetual fear of breast cancer recurrence. They also receive higher doses of systemic chemotherapy and/or radiotherapy which induce inhibiting physical side effects such as alopecia and sleep disruption. The combination of recurrence fear and physical side effects could be directly responsible for women’s diminished ability to carry out their ‘normal’ daily activities such as socialising with friends or attending work.

Moreover, we identified time since diagnosis to be a predictive factor of survivor’s overall quality of life, with a longer period of time since diagnosis corresponding to a lower perceived quality of life. Conceivably, our study found this novel result as women with a longer time since their diagnosis are more likely to experience more intense ruminative thinking regarding the possibility of cancer recurrence. Women with a longer time since diagnosis are also more likely to have come to terms with the long-term detrimental effects that receiving a breast cancer diagnosis and undergoing anti-cancer treatment have on their life (i.e. long-term fatigue induced by treatment might cause social isolation). These findings are highly substantial, not only as they demonstrate predictive links but also as they reiterate the importance of acknowledging time since diagnosis, grade and their effects on breast cancer survivor’s overall well-being in future research.

As a collective, our findings have vital implications for researchers and health care services; in particular, they indicate that further research should be carried out to explore the effects of CCT on the perceived cognitive function, emotional well-being and quality of life of younger breast cancer survivors. As it stands currently, women with a breast cancer diagnosis are not routinely offered any form of CCT, despite strong evidence showing that it profoundly improves emotional well-being (Course-Choi et al., 2017; Motter et al., 2016; Sari et al., 2016; Swainston and Derakshan, 2018).

### Research limitations and future directions for research

The present study displayed some methodological limitations that need to be taken into consideration when interpreting our results. A particular limitation within our study was that the questionnaires were only implemented at the one-time point, meaning that answers could have been influenced by mood or unusual events (i.e. cancer recurrence scare) that may have changed the participants’ current psychological state. Assessing trends of cognition and emotions throughout the diagnosis, treatment trajectory and a few years post-treatment would provide us with valuable data on patterns of perceived cognitive function and emotional vulnerability in breast cancer survivors. A second limitation is that we assumed that all of the women recruited had a sound understanding of the clinical characteristics of their breast cancer diagnosis (i.e. type and grade of tumour) and the treatment received. Although clinical characteristics like grade are very well documented in patient’s official diagnostic (hospital) letters and are focal during discussions with breast cancer clinicians, future research should conduct more individual checks (i.e. via interviews with clinicians or pathologist reports) to ensure that all the demographic information reported is correct. A final limitation of our study is that all of the participants were recruited using advertisements placed on social media platforms including Facebook and Twitter. As a consequence, our sample of women might not be representative of the much broader breast cancer population. In the future, researchers should, therefore, recruit survivors through a broader range of sources including patient oncology clinics.

Considering the current findings of our study, recommendations for future research can be made. We suggest that this study is replicated on a much larger scale to provide a more in-depth account of younger breast cancer survivors’ cognitive and emotional health concerning specific demographic factors and moderator variables. In addition, future studies may wish to adopt a mixed methods approach incorporating qualitative, quantitative, and objective measures, as well as implementing longitudinal studies. This would provide a more thorough personal portrayal of breast cancer survivor’s experience and would also permit for assessing the long-term impact a breast cancer diagnosis and its treatment may have on survivor’s cognitive and emotional health. Finally, given the high association between cognition and emotional vulnerability in this sample, we advocate that future research and clinicians should further explore CCT in the breast cancer population to evaluate its effectiveness as this would aid the development of new interventions that primarily aim to improve emotional resilience in breast cancer survivors.

## Conclusion

This study bestows crucial and novel findings that highlight the detrimental effects that perceived cognitive function, as well as various demographic (risk) factors, can have on breast cancer survivor’s emotional vulnerability and quality of life. Vitally, we established that perceived cognitive function significantly relates to the severity of emotional vulnerability endured by breast cancer survivors with grade of breast cancer moderating this relationship. Considering the shortfall of obtainable psychological interventions at an individual level, our findings are highly pertinent for clinicians and health care settings treating individuals with psychological concerns as a repercussion of a breast cancer diagnosis and its treatment.

## Supplemental Material

Supplement_Table – Supplemental material for Perceived cognitive functioning and its influence on emotional vulnerability in breast cancer Click here for additional data file. Supplemental material, Supplement_Table for Perceived cognitive functioning and its influence on emotional vulnerability in breast cancer by Bethany Chapman, Stefanie Helmrath and Nazanin Derakshan in Health Psychology Open

## Notes

1. Based on the strong positive correlations identified between the Revised Impact of Events Scale (IES-R), Hospital Anxiety and Depression Scale (HADS) and Cancer Worry Scale (CWS) (all *r*s > .30, all *p* < .01), the standardised total scores achieved on these three questionnaires were combined for all subsequent analyses and are referred to as ‘emotional vulnerability’.
