# Supplementary material for: Perceived cognitive functioning and its influence on emotional vulnerability in breast cancer
Source: Health Psychol Open. 2019 Aug 23;6(2):2055102919871661. doi: 10.1177/2055102919871661 (PMC6710691; doi:10.1177/2055102919871661)
Supplement: Supplement_Table – Supplemental material for Perceived cognitive functioning and its influence on emotional vulnerability in breast cancer [file Supplement_Table.pdf]

Table 1 Participants' Breast Cancer Specific Clinical History, Psychological and Neurological Medical History (N = 132)

		Number of Participants (%)
Diagnosis of Breast Cancer	Primary Breast Cancer	119 (90.2)
	Secondary Breast Cancer	12 (9.1)
	Stage of Breast Cancer Unreported	1 (0.8)
Type of Breast Cancer Diagnosed	Ductal carcinoma in Situ (DCIS)	21 (15.9)
	Invasive Ductal Carcinoma (IDC)	53 (40.2)
	Invasive Ductal Carcinoma (IDC) with Metaplastic	1 (0.8)
	Mixed IDC and DCIS	29 (22.0)
	Mixed IDC and DCIS with Cribiform Breast Cancer	1 (0.8)
	Invasive Lobular Breast Cancer	14 (10.6)
	Inflammatory Breast Cancer	6 (4.5)
	Metaplastic Breast Cancer	1 (0.8)
	Ductal Carcinoma in Situ (DCIS) with Invasive Lobular	1 (0.8)
	Mixed Ductal and Lobular	1 (0.8)
	Inflammatory Breast Cancer with Mediastinal Nodal Disease	1 (0.8)
	Other	2 (1.5)
	Type Unreported	1 (0.8)
Grade of Tumour	Low (Grade One)	18 (13.6)
	Moderate (Grade Two)	52 (39.4)
	High (Grade Three)	59 (44.7)
	Grade Unreported	3 (2.3)

Lymph Nodes		
Involvment	Yes	76 (57.6)
	No	54 (40.9)
	Involvement Unknown	2 (1.5)
Quantity of Lymph		
Nodes Involved	No Lymph Nodes	53 (40.2)
	1-5	48 (36.4)
	6-10	9 (6.8)
	11+	12 (9.1)
	Quantity Unknown	6 (4.5)
	Unreported	4 (3.0)
Hormone Receptor		
Status	ER+PR+Her2+	11 (8.3)
	ER+PR+Her2-	46 (34.8)
	ER+PR-Her2+	4 (3.0)
	ER+PR-Her2-	29 (22.0)
	ER-PR+Her2-	2 (1.5)
	ER-PR-Her2+	11 (8.3)
	ER-PR-Her2-	13 (9.8)
	Receptor Status Unknown	1 (0.8)
	Unreported	15 (11.4)
Treatment Received	Neo-adjuvant Chemotherapy, Lumpectomy & Radiotherapy	15 (11.4)

	Neo-adjuvant Chemotherapy, Mastectomy & Radiotherapy	24 (18.2)
	Neo-adjuvant Chemotherapy & Mastectomy	7 (5.3)
	Lumpectomy, Chemotherapy & Radiotherapy	20 (15.2)
	Mastectomy, Chemotherapy & Radiotherapy	16 (12.1)
	Mastectomy & Chemotherapy	14 (10.6)
	Lumpectomy & Radiotherapy	17 (12.9)
	Mastectomy	18 (13.6)
	Lumpectomy, Radiotherapy & Mastectomy	1 (0.8)
Class of Mastectomy Procedure	Uni-lateral	55 (41.7)
	Bi-lateral	24 (18.2)
	No Mastectomy	49 (37.1)
	Unreported	4 (3.0)
Cycles of Herceptin Injections Received	9 cycles	0 (0)
	18 cycles	21 (15.9)
	Complete Herceptin Cycles	1 (0.8)
	No Herceptin Cycles Received	110 (83.3)
Endocrine Therapies Administered	Yes	93 (70.5)
	No	39 (29.5)
Class of Endocrine Therapies Received	Tamoxifen	43 (32.6)
	Zoladex	3 (2.3)

	Aromatase Inhibitor	28 (21.2)
	Zoladex & Tamoxifen	3 (2.3)
	Zoladex & Aromatase Inhibitor	12 (9.1)
	Prescribed but not taken by the Participant	3 (2.3)
	Type of Therapies Unreported	1 (0.8)
	No Endocrine Therapy Received	39 (29.5)
Diagnosis of Psychiatric Disorder	Yes	44 (33.3)
	No	88 (66.7)
Diagnosed Psychiatric Condition	Depression	28 (21.2)
	Anxiety	5 (3.8)
	Depression & Anxiety	8 (6.1)
	Post-traumatic stress distress (PTSD) with or without Depression	2 (1.5)
	Psychosis	1 (0.8)
	No Condition	88 (66.7)
Clinical Diagnosis of a Neurological Condition	Yes	8 (6.1)
	No	123 (93.2)
	Unreported	1 (0.8)
Diagnosed Neurological Condition	Epilepsy	2 (1.5)
	Migraine	1 (0.8)
	Vulvodynia	1 (0.8)

Fibromyalgia	1 (0.8)
Myalgic Encephalomyelitis (M.E.)	2 (1.5)
Neuropathy	1 (0.8)
No condition	124 (93.9)
